# The actual knee function was not influenced by joint line obliquity after open-wedge high tibial osteotomy

**DOI:** 10.1051/sicotj/2020001

**Published:** 2020-01-31

**Authors:** Mitsuaki Kubota, Youngji Kim, Taisuke Sato, Junichiro Yamaguchi, Ryuichi Ohno, Kazuo Kaneko, Muneaki Ishijima

**Affiliations:** 1 Department of Orthopaedic Surgery, Koshigaya Municipal Hospital 10-47-1, Higashi-Koshigaya Saitama 3430023 Japan; 2 Department of Orthopaedic Surgery and Spoerts Medicine, Juntendo University, School of Medicine 1-2-1 Hongo, Bunkyo-ku Tokyo 1138421 Japan

**Keywords:** open-wedge high tibial osteotomy (OWHTO), joint line obliquity, patient-reported outcome, actual knee function

## Abstract

*Purpose*: Excessive joint line obliquity (JLO) after open-wedge high tibial osteotomy (OWHTO) induces detrimental stress on the articular cartilage. The purpose of this article is to assess the correlation between JLO and the clinical results after OWHTO.

*Methods*: 68 patients were followed up for more than 1 year. JLO was assessed using a long-leg standing anteroposterior radiograph. The knee osteoarthritis outcome score (KOOS) and KSS (Knee Society score) objective knee score were assessed as clinical scores. The Weight-bearing line ratio (WBLR), medial proximal tibial angle (MPTA), lateral distal femoral angle (LDFA), and joint line convergence angle (JLCA) were assessed as radiological parameters. The timed up-and-go (TUG) test and single-leg standing (SLS) test were performed, and the isometric muscle strength of the quadriceps and hamstrings was assessed to evaluate the knee function. The primary outcomes were the correlations between the JLO and the clinical score, radiological parameters and knee function after OWHTO. The secondary objective of this study was to detect the factor with the greatest influence on JLO.

*Results*: There were significant correlations between the postoperative JLO and the KOOS in the subcategories of pain, activities of daily living (ADL), and sports and recreation (*r* = −0.311, −0.302, −0.282, *p* = 0.011, 0.014, 0.022, respectively). However, the postoperative JLO was not significantly correlated with the KSS, knee function, or muscle strength. The preoperative LDFA and postoperative MPTA were factors influencing increased JLO after OWHTO.

*Discussion*: There was no significant correlation between the JLO and the actual knee function. The preoperative LDFA and postoperative MPTA were factors that influenced the increase in JLO after OWHTO.

## Introduction

Knee joint line obliquity (JLO) after open-wedge high tibial osteotomy (OWHTO) corresponds to an excessive valgus of the tibial mechanical axis [1]. To resolve these problems for severe varus alignment, the proximal tibia should be overcorrected through OWHTO, which can increase the JLO in the coronal plane. Excessive JLO increases the shear stress at the joint surface [1–5]. Over 5° of JLO may induce detrimental stress on the articular cartilage [3]. JLO should be parallel to the floor in the coronal plane to prevent further progression of osteoarthritis. Varus alignment does not affect the joint line orientation. Advanced medial arthritis causes divergence of the joint line from parallel to the floor. These findings influence decision-making regarding osteotomy and alignment in total knee arthroplasty [6].

Although one report has compared the clinical results and JLO after HTO [7], it remained unclear whether there was no significant correlation between the clinical results and JLO in inactive patients, while a significant correlation was found in active patients who performed running, jumping, twisting, and kneeling in sports and recreational activities. We hypothesized that there would be no significant correlation between JLO and the clinical results of inactive patients, while a significant correlation would be observed in active patients. In addition, this study assessed the correlations between JLO and radiological parameters. We also hypothesized that a preoperative large distal femoral angle (LDFA) and a postoperative large medial femoral angle (MPTA) would be correlated with the JLO after OWHTO.

## Patients and methods

From November 2011 to March 2018, 75 consecutive patients with primary medial osteoarthritis were treated with high tibial osteotomy. Patients with symptomatic osteoarthritis of the patellofemoral joint and lateral compartment, rheumatoid arthritis, a knee range of motion of < 100°, high-grade ligamentous instabilities, and extensive loss (or the absence) of the lateral meniscus were excluded from the study.

Preoperative and postoperative radiographs were obtained prospectively in all cases. We prospectively evaluated the radiographs and the clinical results of all cases. Patients for whom radiographs were obtained preoperatively and one year after OWHTO were selected. Five patients were excluded due to other procedures (tibial condylar valgus osteotomy [TCVO]: *n* = 2; and hybrid closed wedge HTO: *n* = 3). Two patients were excluded due to being lost to follow-up. One 75-year-old woman was excluded because she developed Guillain-Barre syndrome at 10 months after OWHTO. Thus, a total of 68 cases were evaluated in this study.

### Clinical and radiological evaluations

The knee osteoarthritis outcome score (KOOS) was examined as a patient-reported outcome. All KOOS subcategories, namely, pain, symptoms, activities of daily living (ADLs), sports and recreation, and quality of life (QOL), were evaluated. In addition, the KSS (Knee Society Score) objective knee score was examined as an objective clinical assessment for all patients [8]. The timed up-and-go (TUG) test and single-leg standing (SLS) test were performed as assessments of the knee function. Furthermore, the isometric muscle strength of the quadriceps and hamstrings was measured. All examinations were performed for all patients before and after OWHTO.

**Figure 1:**
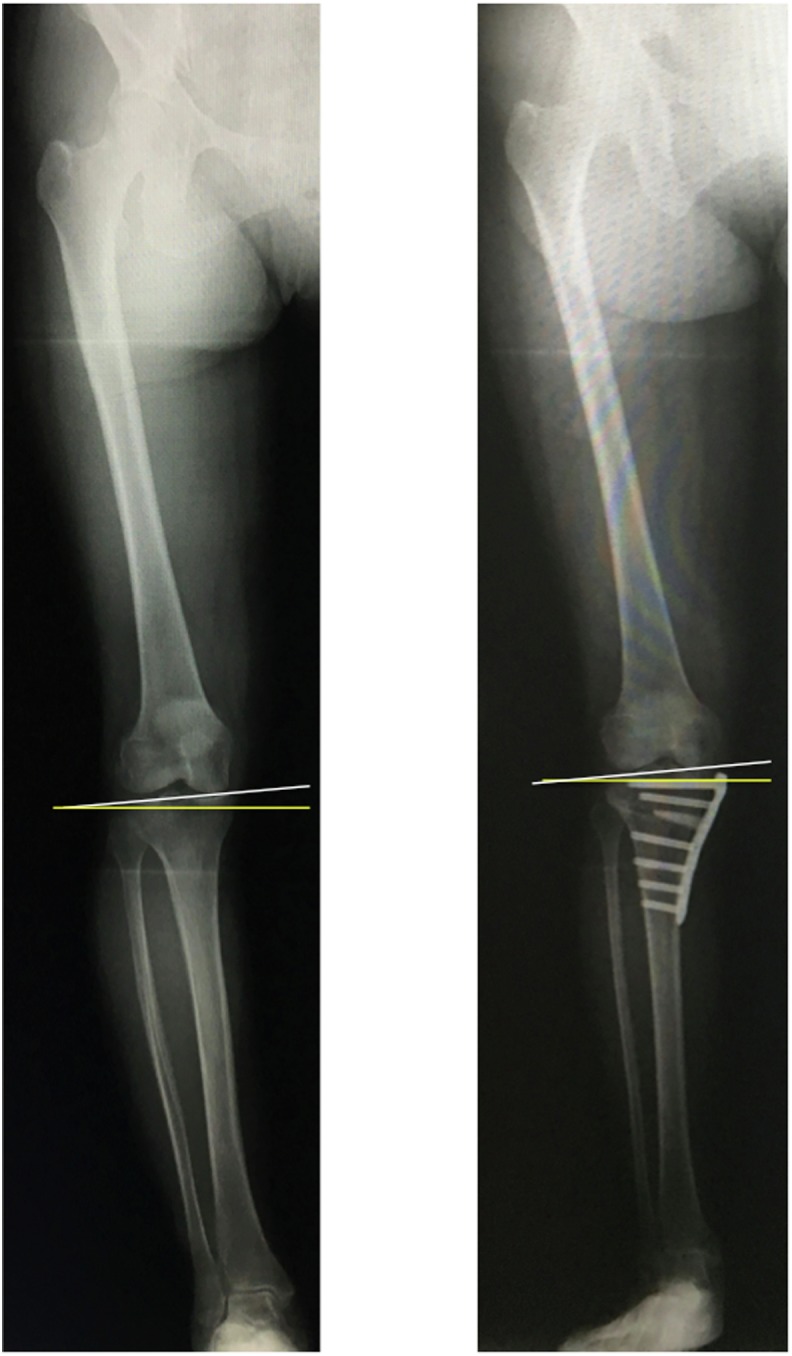
Joint line obliquity. Single-limb standing anteroposterior radiograph of a lower extremity. JLO was defined as the angle between the line parallel to the ground (yellow line) and the articular surface of the proximal tibia (white line). Medial inclination was defined as “+” and lateral inclination was defined as “–”.

**Figure 2:**
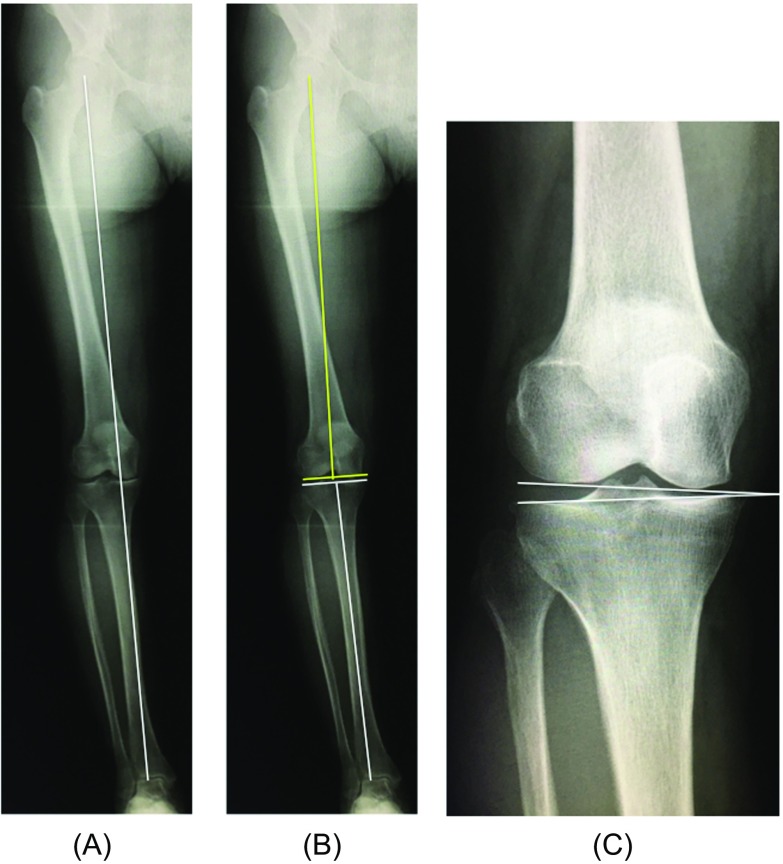
Radiological parameters. (A) The weight-bearing line (WBL) was defined as the line drawn from the center of the femoral head to the center of the superior articular surface of the talus. The WBL ratio was defined as the tibial insertion of the weight-bearing line/tibial width, with the medial tibial edge at 0% and the lateral tibial edge at 100%. (B) The mechanical lateral distal femoral angle (LDFA) was defined as the angle between the femoral mechanical axis and the articular surface of the distal femur (yellow line). The medial proximal tibial angle (MPTA) was defined as the angle between the tibial mechanical axis and the articular surface of the proximal tibia (white line). (C) The joint line convergence angle (JLCA) was defined as the angle of the two articular surface lines of the distal femur and the proximal tibia.

An anteroposterior long-axis radiograph under single-limb standing was also taken for all patients before and after surgery. The JLO was measured as a radiological parameter (Figure 1) as the angle formed by the line parallel to the ground and the line tangent to the tibial condyles (medial inclination: +, lateral inclination: −) [9]. The Weight-bearing line ratio (WBLR), mechanical LDFA, mechanical MPTA, and JLCA were also measured as radiological parameters (Figure 2).

The intra- and inter-observer reliabilities of each measurement were assessed by determining the intraclass correlation coefficient (ICC). The radiological parameters were measured by two orthopedic surgeons (M.K and Y.K.; independent observers who were blinded to other data), twice at an interval of 2 weeks.

### Institutional review board approval

Informed consent for the use of medical data was obtained from all patients, and this study was approved by the institutional review board of Koshigaya Municipal Hospital (Approval no. 30-5).

### Statistical analysis

The primary outcomes were the correlations between the JLO and the clinical results and radiological parameters. Spearman’s correlation coefficients were determined to analyze correlations. The secondary objective was to determine which of the following factors was the most predictive of JLO: preoperative and postoperative WBLR, MPTA, LDFA, and JLCA. A multiple linear regression analysis was performed to evaluate these associations. All statistical analyses were performed using IBM SPSS Statistics version 22 software program (IBM Corporation, Armonk, NY, USA). A post hoc analysis of the correlation between the alignment error and the other radiographic parameters was performed to determine the statistical power using the G*Power software program (version 3.1; Heinrich Heine, Düsseldorf, Germany). The statistical power was 0.95 with an effect size of 0.5, an alpha value of 0.05, and a sample size of 42. *P* values of < 0.05 were considered to indicate statistical significance. All data are presented as the mean value and standard deviation.

## Results

The average age at surgery was 60.3 years (range: 37–78), the mean BMI was 26.0 (range: 19.7–37.8), and the average follow-up period was 2.5 years (range: 1–7). All KOOS subcategory and KSS objective knee score were significantly improved; the knee function and muscle strength were also significantly improved (Table 1). The postoperative JLO, WBLR, and MPTA were significantly higher than their corresponding preoperative values (*p* < 0.001, respectively). The preoperative and postoperative LDFA and JLCA did not differ to a statistically significant extent (Table 1). The inter-observer and intra-observer reliabilities in the assessment of the radiographic parameters were almost satisfactory (Inter-observer reliability: JLO = 0.859, WBLR = 0.893, LDFA = 0.910, MPTA = 0.980, JLCA = 0.797; Intra-observer reliability: JLO = 0.910, WBLR = 0.869, LDFA = 0.970, MPTA = 0.971, JLCA = 0.911).

**Table 1:** The preoperative and postoperative clinical and radiological results.

	Pre-operation	Post-operation	*p* Value
KOOS Pain	49.1 ± 19.4	80.0 ± 15.1	0.000*
KOOS Symptoms	59.3 ± 19.8	78.3 ± 15.5	0.000*
KOOS ADL	63.5 ± 16.2	85.5 ± 13.9	0.000*
KOOS Sports and Recreation	26.6 ± 17.8	55.2 ± 26.3	0.000*
KOOS QOL	27.2 ± 14.7	60.1 ± 24.7	0.000*
KSS	65.8 ± 5.8	92.6 ± 7.0	0.000*
TUG (s)	9.6 ± 2.6	7.9 ± 1.3	0.000*
SLS (s)	19.5 ± 11.7	24.0 ± 9.8	0.003*
Quadriceps strength (%BW)	110.4 ± 50.0	144.6 ± 51.6	0.000*
Hamstrings strength (%BW)	43.3 ± 21.0	74.6 ± 27.9	0.000*
JLO (°)	1.1 ± 3.3	2.6 ± 2.8	0.001*
WBLR (%)	14.2 ± 12.1	64.2 ± 19.5	0.000*
LDFA (°)	88.7 ± 2.2	88.3 ± 2.0	0.673
MPTA (°)	84.9 ± 2.5	93.7 ± 3.0	0.000*
JLCA (°)	3.4 ± 2.2	2.6 ± 2.0	0.795

Data are shown as the mean (standard deviation). KOOS: knee osteoarthritis outcome score; KSS: knee society score; TUG: timed up-and-go test; SLS: single-leg standing test; Quadriceps strength: % body weight of isometric muscle strength; JLO: joint line obliquity; WBLR: weight-bearing line ratio; LDFA: mechanical lateral distal femoral angle; MPTA: mechanical medial proximal tibial angle; JLCA: joint line convergence angle.

* Statistically significant difference in comparison to pre-operation.

The primary outcomes are summarized in Table 2. Significant correlations were noted between the JLO and KOOS subcategories of pain, ADL, and sports and recreation in the clinical results after OWHTO (*r* = −0.311, −0.302, −0.278, *p* = 0.011, 0.014, 0.022, respectively). However, no significant correlations were observed between JLO and the KSS, TUG, SLS, or muscle strength. Regarding the radiological results, the preoperative WBLR, LDFA, and postoperative LDFA and MPTA were significantly correlated with postoperative JLO (*r* = −0.294, 0.539, 0.405, 0.554, *p* = 0.017, 0.000, 0.001, 0.000, respectively). In particular, the preoperative LDFA and postoperative MPTA were strongly correlated with postoperative JLO.

**Table 2:** Factors correlated with joint line obliquity after OWHTO.

	*r*	*p* Value
KOOS Pain Post	−0.311	0.011*
KOOS Symptom Post	−0.228	0.065
KOOS ADL Post	−0.302	0.014*
KOOS Sports and Recreation Post	−0.278	0.022*
KOOS QOL Post	−0.213	0.086
KSS Post	0.049	0.699
TUG (s) Post	0.076	0.543
SLS (s) Post	−0.160	0.198
Quadriceps strength (%BW)	−0.116	0.198
Hamstrings strength (%BW)	−0.081	0.559
WBLR (%) Pre	−0.294	0.017*
WBLR (%) Post	0.044	0.726
LDFA (°) Pre	0.539	0.000*
LDFA (°) Post	0.405	0.001*
MPTA (°) Pre	0.226	0.070
MPTA (°) Post	0.554	0.000*
JLCA (°) Pre	0.240	0.056
JLCA (°) Post	0.044	0.726

OWHTO: open wedge high tibial osteotomy; KOOS: knee osteoarthritis outcome score; KSS: knee society score; TUG: timed up-and-go test; SLS: single leg standing test; Quadriceps strength: % body weight of isometric muscle strength; WBLR: weight-bearing line ratio; LDFA: mechanical lateral distal femoral angle; MPTA: mechanical medial proximal tibial angle; JLCA: joint line convergence angle.

* Statistically significant difference.

**Figure 3:**
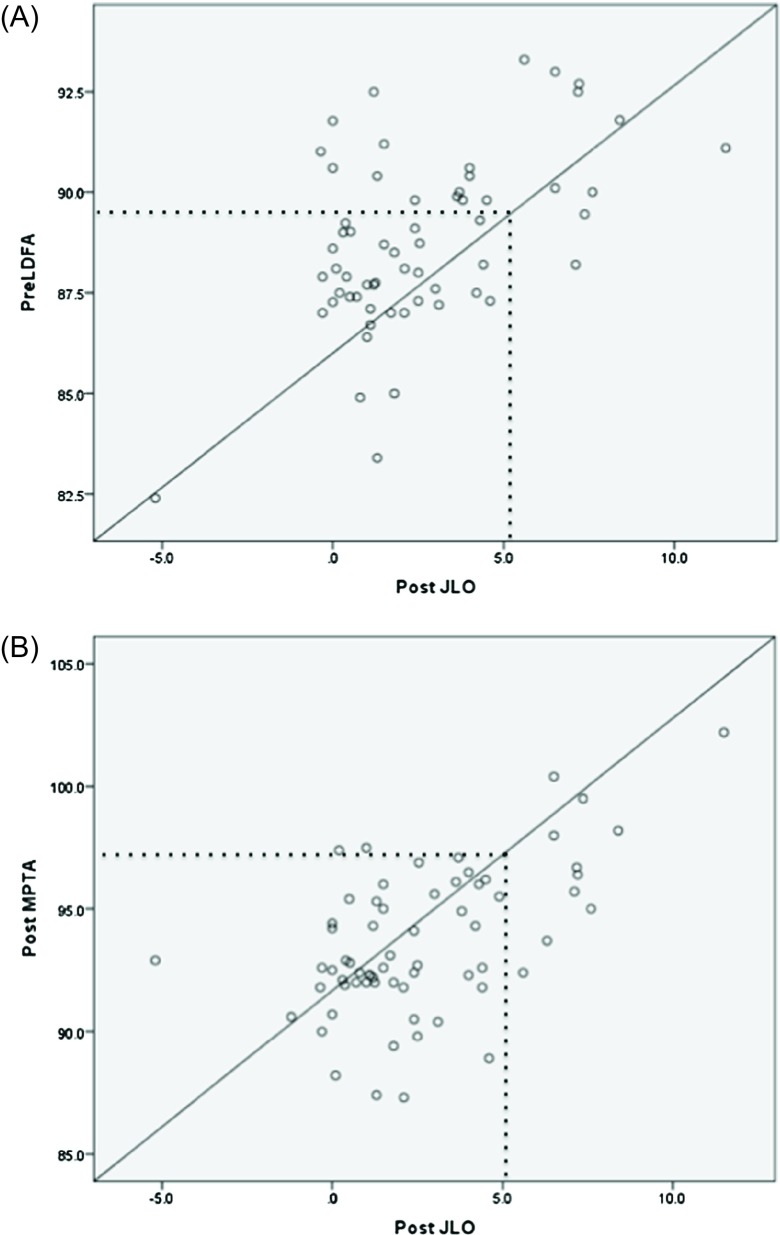
The correlations between post-operative JLO and pre- and post-operative radiological parameters. (A) The correlations between post-operative JLO and pre-operative LDFA. 5° of postoperative JLO corresponds to a preoperative LDFA of 90° (dotted line). (B) The correlations between post-operative JLO and post-operative MPTA. 5° of postoperative JLO corresponds to a postoperative MPTA of 97.5° (dotted line).

The secondary outcomes are shown in Table 3. A multiple linear regression analysis indicated that, among the preoperative and postoperative WBLR, LDFA, MPTA, and JLCA, the preoperative LDFA and postoperative MPTA were associated with increased JLO after OWHTO (*β* = 0.321, 0.333; *R* ^2^ = 0.460; *p* = 0.030, 0.025, respectively). These results indicated that a higher preoperative LDFA and a higher postoperative MPTA resulted in increased JLO. The Spearman’s correlation analysis revealed that 5° of postoperative JLO corresponds to a preoperative LDFA of 90° and a postoperative MPTA of 97.5° (Figures 3A and 3B).

**Table 3:** Multiple linear regression analysis of factors associated with joint line obliquity after OWHTO.

	*β*	*R* ^2^	*p* Value
WBLR Pre	−0.177	0.460	0.214
WBLR Post	0.062		0.577
LDFA Pre	0.321		0.030*
LDFA Post	0.067		0.619
MPTA Pre	0.103		0.479
MPTA Post	0.333		0.025*
JLCA Pre	−0.003		0.983
JLCA Post	0.196		0.107

OWHTO: open wedge high tibial osteotomy; WBLR: weight-bearing line ratio; LDFA: mechanical lateral distal femoral angle; MPTA: mechanical medial proximal tibial angle; JLCA: joint line convergence angle.

* Statistically significant difference.

## Discussion

The most important finding of this study was that there were no significant correlations between JLO and objective assessments of the knee, the actual knee function, or muscle strength. However, the JLO was significantly correlated with patient-reported outcomes with regard to pain, ADLs, and sports and recreation activities. Our hypothesis, that there would be no significant correlation between the JLO and the clinical results was therefore half affirmed, and half denied.

Single-level osteotomy of the tibia with severe varus deformity requires a large amount of correction, which results in non-anatomical JLO [1, 5, 10–13]. Non-anatomical JLO after HTO may induce several problems, including increased shear force at the knee and femoral subluxation [14–16]. However, there were no significant problems in patients with high JLO after HTO, with the exception of patient-reported outcome measures. It is considered that the actual knee function was not affected by JLO. Coventry suggested that up to 10° of JLO is acceptable after correction osteotomy [17]. In contrast, Babis advocated that the maximal JLO should not exceed 4° [1]. The correlation between JLO and the clinical results was recently reported [7]. Patients with a postoperative MPTA of > 95° had greater valgus alignment and JLO with a lower KOOS subscale scores for sports and recreation than those with a postoperative MPTA of ≤ 95° at 2 years after surgery. Furthermore, the American Knee Society function scores of the two groups did not differ to a statistically significant extent. The results were similar to our results.

Double-level osteotomy (DLO) is a useful procedure for patients with severe varus deformity preserving an acceptable JLO which cannot be achieved by a single-level procedure [1, 12]. Over 5° of JLO may induce detrimental stress to the articular cartilage [3]. In the radiological assessment, the preoperative LDFA and postoperative MPTA were associated with postoperative JLO. The correlation analysis revealed that 5° of postoperative JLO corresponded to a preoperative LDFA of 90°and a postoperative MPTA of 97.5°. However, is it better to perform DLO for all cases that exceed this angle? The mean ages of patients who underwent DLO in these two previous studies were 50.1 years and 50.9 years, respectively (range: 20–65 years) [1, 12]. In contrast, the mean age of the patients in our study was 60.3 years (range: 37–78 years); thus, our patients were older than those who underwent DLO in previous reports. This may explain the lack of any significant correlation between JLO and the knee function, as assessed in our study, as DLO is recommended for active patients of < 60 years of age.

There was divergence between the patient-reported outcomes, the knee function assessments, and the actual knee function in our study. It was considered that knee function was not reflected in the results because the average follow-up period was too short. Moreover, the JLO was not significantly correlated with the knee function, because the TUG test and SLS test were easy to perform for all patients. Another knee function test may demonstrate a significant correlation between JLO and the knee function.

JLO was correlated with the preoperative WBL, LDFA, and the postoperative LDFA and MPTA after OWHTO. Oh et al. reported that the preoperative hip-knee-ankle angle (HKA) and JLCA were predictors of abnormal JLO (>4°) after OWHTO [5]. The odds ratio for abnormal JLO was 1.27 for the preoperative HKA and 2.13 for the preoperative JLCA. However, the postoperative radiological parameters were not correlated with the JLO. The results of our study were similar with regard to the preoperative WBLR but not with regard to postoperative parameters. A higher preoperative LDFA and higher postoperative MPTA were associated with greater JLO after OWHTO. A higher MPTA equates to excessive correction during surgery, and excessive correction in OWHTO can cause an increase in JLO; thus, surgeons should be careful not to overcorrect when treating active patients.

The present study was associated with several limitations. First, the ankle joint obliquity was not mentioned. A long lever arm through the tibia can cause greater changes in ankle joint line obliquity than in JLO after OWHTO [18]. However, it was reported that ankle joint parameters were not significant predictors of abnormal JLO after OWHTO [5]. Second, this study mentioned only the coronal plane of the lower limb alignment. Malalignment of the lower limb in the sagittal or axial planes may cause a correction error. Bony deformity, which can also cause limb alignment correction error, was not addressed in this study [19, 20]. Finally, the study participants were relatively few. The inclusion of a greater number of cases may prove that the postoperative MPTA is a strong predictor of JLO after OWHTO.

JLO was not significantly associated with the results of knee function assessments or the actual knee function. Thus, DLO should not be performed in all patients with severe varus alignment. Rather, the indication of DLO should be limited to young, active patients.

## Conclusions

Significant correlations were observed between JLO and patient-reported outcomes; however, JLO was not significantly associated with the results of knee function assessments or the actual knee function. The preoperative LDFA and postoperative MPTA were correlated with the JLO after OWHTO.

## Conflict of interest

The authors declare that they have no conflict of interest and received no funding for this work.

## Ethical approval

All procedures performed in studies involving human participants were in accordance with the ethical standards of the institutional and national research committee and with the 1964 Helsinki Declaration and its later amendments or comparable ethical standards.

## Informed consent

Informed consent was obtained from all individual participants included in the study.
